# A Multi‐Centre, Randomised, Controlled Clinical Trial Assessing Cryopreserved Ultra‐Thick Human Amniotic Membrane in the Treatment of Complex Diabetic Foot Ulcers

**DOI:** 10.1111/wrr.70110

**Published:** 2025-12-04

**Authors:** Joseph Caporusso, Travis Motley, John C. Lantis, Stephen Heisler, Adam Hicks, Stephanie C. Wu, Alexander Reyzelman

**Affiliations:** ^1^ Futuro Clinical Trials McAllen Texas USA; ^2^ Acclaim Physician Group John Peter Smith Hospital Fort Worth Texas USA; ^3^ Department of Surgery Icahn School of Medicine New York New York USA; ^4^ Department of Surgery Mount Sinai West Hospital New York New York USA; ^5^ Department of Surgery University of North Carolina School of Medicine Chapel Hill North Carolina USA; ^6^ Podiatric Medicine and Surgery, Department of Orthopaedic Surgery Vanderbilt University Medical Center Nashville Tennessee USA; ^7^ Department of Podiatric Surgery and Applied Biomechanics and Center for Stem Cell and Regenerative Medicine William M. Scholl College of Podiatric Medicine, Rosalind Franklin University of Medicine and Science Chicago Illinois USA; ^8^ College of Podiatric Medicine, Samuel Merritt University Oakland California USA; ^9^ Center for Limb Preservation and Diabetic Foot, Division of Vascular and Endovascular Surgery University of California San Francisco San Francisco California USA

**Keywords:** amniotic membrane, amputation, birth tissue, diabetic foot ulcer, umbilical cord

## Abstract

TTAX01/Neox 1K are cryopreserved ultra‐thick human amniotic membrane products derived from umbilical cord (cUC) that have been assessed for clinical effectiveness in complex diabetic foot ulcers (DFUs). Herein, a randomised controlled trial was conducted to assess the safety and efficacy of cUC versus standard of care (SOC) for DFUs with exposed bone, tendon, muscle and/or joint capsule and controlled osteomyelitis. A total of 220 eligible patients were enrolled and randomised to receive cUC + SOC (*n* = 118) or SOC alone (*n* = 102), which included debridement, bone resection, wound dressings, offloading and a 6‐week course of systemic antibiotics. cUC was applied at baseline and reapplied at a minimum of 4‐week intervals if healing was stalled throughout a 16‐week treatment period, for a maximum of four applications. The mean baseline wound area for the cUC and SOC groups was 5.64 ± 5.5 cm^2^ and 5.30 ± 4.6 cm^2^, respectively. By 26 weeks, 139 patients achieved complete healing in the intent‐to‐treat population (66.1% cUC group vs. 59.8% SOC group; *p* = 0.40). An average of 1.67 ± 0.87 applications were required to achieve wound closure in the cUC group. By 50 weeks, 77.1% of patients treated with cUC achieved complete healing compared to 71.6% in the SOC group (*p* = 0.29). Adverse event rates, i.e., 89.8% and 87.3%, were comparable between cUC and SOC groups. While there were no significant differences in healing rates or adverse events between the two treatment arms at any time point, this study demonstrates that adjunctive cUC is safe and helps achieve a high healing rate at 50 weeks with less than four applications for complex DFUs that are often excluded in clinical trials.

**Abbreviations**
ABI: ankle brachial index
AE: adverse event
AM: amniotic membrane
BMI: body mass index
CGMP: current good manufacturing practices
CGPT: current good tissue practices
CI: confidence interval
cUC: cryopreserved umbilical cord
DFU: diabetic foot ulcer
HbA1c: haemoglobin A1c
HC‐HA/PTX3: heavy chain‐hyaluronic acid/pentraxin 3
HR: hazard ratio
ICH: International Conference on Harmonisation
IRB: Institutional Review Board
ITT: intent‐to‐treat
LEA: lower extremity amputation
MRI: magnetic resonance imaging
RCT: randomised controlled trial
SOC: standard of care
TcPO_2_
: transcutaneous oxygen pressure
UC: umbilical cord

## 1 Introduction

In 2019, the International Diabetes Federation reported that diabetes affected a staggering 463 million people—a number that is expected to rise to 700 million by 2045 [1]. People with diabetes are at risk for serious complications, the most significant being a diabetic foot ulcer (DFU). It is estimated that 25% of the diabetic population will develop a DFU during their lifetime, with an estimated annual incidence rate of 0.5%–3.0% [2, 3, 4, 5, 6, 7]. These statistics are alarming, as the 5‐year mortality rate for diabetic patients with a DFU ranges from 31% to 40% [8, 9]. Moreover, a considerable number of DFU patients require amputation each year, which significantly reduces quality of life, exacerbates the social burden, and shortens life expectancy [10, 11, 12]. In fact, 50%–70% of all lower extremity amputations (LEA) are caused by DFUs, with more than 73,000 LEAs performed in the United States for patients with diabetes each year [13, 14].

Three major risk factors have been recognised to complicate non‐healing DFUs and contribute to limb amputation: ulcer depth, infection, and ischemia [15]. The first factor, ulcer depth, is of particularly high importance, as a vast number of studies have shown that deep, complex ulcers with exposed bone, tendon, joint, or muscle are at a significantly high risk of undergoing amputation [12, 16, 17, 18, 19, 20]. When considering major amputations, several studies have shown that Wagner grade 1 and 2 DFUs result in an amputation rate of 3%–4% compared to 32%–38% for Wagner grade 3 and 4 DFUs [19, 21]. This is problematic as the risk of mortality increases 2.8 times in diabetic patients that undergo major amputation, with a 5‐year mortality rate of 56.6% [8, 10, 11, 12]. Unsurprisingly, the financial burden of DFUs is cumbersome, with national costs exceeding those of non‐DFU care by more than 10‐fold [22]. DFU care costs the healthcare system approximately $3000–$108,000 per ulcer depending on the extent and management of disease [23]. Despite these high costs, 55% of DFUs fail to heal [24]. This healing rate is even lower for complex DFUs with higher Wagner grades, with 33% of ulcers achieving complete wound closure within 20‐weeks [25]. As a result, advanced therapies have become increasingly used to improve healing rates, reduce the risk of amputation and ultimately decrease treatment costs.

Amniotic membrane (AM) and ultra‐thick AM derived from umbilical cord (UC) tissues have been increasingly used in the management of chronic, non‐healing wounds due to their unique wound healing properties [26, 27, 28]. Notably, both AM and UC have been shown to contain anti‐inflammatory and anti‐scarring properties that aid in regenerative wound healing, which are attributed in part to a key matrix component present in both AM and UC called heavy chain‐hyaluronic acid/pentraxin 3 (HC‐HA/PTX3) [29, 30]. HC‐HA/PTX3 reduces inflammation by promoting apoptosis of activated neutrophils and macrophages [31, 32, 33, 34] and polarising macrophages towards the M2 phenotype [32, 34]. Additionally, HC‐HA/PTX3 reduces scarring by downregulating transforming growth factor‐β (TGF‐β) promoter activity and preventing myofibroblast differentiation [31, 35, 36, 37, 38, 39]. While AM and UC tissues can be processed utilising various methods, cryopreservation has been shown to better preserve and retain HC‐HA/PTX3 compared to other processing methods [40]. Furthermore, when compared to AM tissue, UC contains significantly higher amounts of HC‐HA/PTX3 and is 10 times thicker [40], making it an advantageous wound covering for deep ulcers with exposed tendon, ligament and bone. Preliminary retrospective studies utilising cryopreserved UC (cUC) (i.e., Neox 1K; BioTissue Holdings Inc., Miami, FL) have demonstrated promising results in chronic DFUs with exposed bone, tendon, muscle and/or joint capsule, with healing rates ranging from 79% to 100% [41, 42]. This data is further supported by a recent prospective trial in which 32 patients with complex DFUs were treated with cUC, resulting in a high proportion of healed wounds (50%) at 16 weeks utilising an average of 1.5 applications. Given these results, this prospective, randomised, controlled study was conducted to assess the safety and efficacy of cUC in patients with complex DFUs with exposure of underlying bone, tendon, muscle and/or joint capsule and medically and surgically controlled osteomyelitis versus standard of care (SOC) alone.

## 2 Materials and Methods

This prospective, multi‐centre, randomised controlled trial (RCT) was conducted across 16 enrolling sites in the United States between June 2020 and July 2024 in accordance with the provisions of the Declaration of Helsinki, International Conference on Harmonisation (ICH) guidelines, and all applicable regulatory requirements. This study was pre‐registered at http://ClinicalTrials.gov (NCT04176120). All participating centres sought review and approval of the study protocol and procedures, study amendments, informed consent form and any recruitment procedures by their designated Institutional Review Board (IRB). All participants provided written informed consent prior to any study‐related procedures.

### 2.1 Trial Design and Participants

The purpose of this study was to determine the safety and efficacy of cUC (BioTissue Holdings Inc., Miami, FL) in the treatment of late‐stage, complex, non‐healing DFUs. All eligible patients were randomised in a 1:1 ratio to receive cUC and SOC or SOC alone with stratification based on the index wound size. Due to the nature of the product and the treatment modality employed, the study was not blinded. Participants were expected to complete weekly visits until Week 19, then biweekly visits until complete wound healing or 26 weeks, whichever came first, then monthly follow‐up visits until Week 50. All patients received SOC throughout the study, with cUC treatment limited to the first 16 weeks. The design of this randomised, controlled study was similar to the design of the open‐label, single‐arm prospective study as previously reported [43] and was performed with continuous enrolment of all eligible patients who wished to participate and provided informed consent.

Patients were eligible for inclusion in the study if they met the following criteria in a maximum of two screening attempts: at least 18 years of age, had confirmed diagnosis of Type I or Type II Diabetes Mellitus, were under the care of a physician for diabetes management, had an index ulcer located on the plantar surface, inter‐digital, heel, lateral, or medial surface of the foot with evidence of exposed bone, tendon, muscle and/or joint capsule, had an index ulcer area ≤ 12.0 cm^2^, had clinical suspicion of osteomyelitis supported by positive probe to bone as well as either radiographic evidence (X‐ray, magnetic resonance imaging [MRI], or bone scan) or evidence of bone necrosis, and had adequate perfusion as determined by ankle brachial index (ABI) ≥ 0.7 to ≤ 1.3 or transcutaneous oxygen pressure (TcPO_2_) ≥ 40 mmHg on the dorsum of the affected foot, or great toe pressure ≥ 50 mmHg. Exclusion criteria included the following: index ulcer primarily located on the dorsal surface of the foot or over an acute Charcot deformity, index ulcer could be addressed by primary closure through the completion of the initial or staged surgical procedure at baseline, glycated haemoglobin A1c (HbA1c) level of > 12%, malignancy or a history of cancer (other than non‐melanoma skin cancer) in the 5 years prior to screening, was taking canaglifozin at the time of screening, received parenteral corticosteroids, oral steroids (> 7.5 mg daily) or any cytotoxic agents for 7 consecutive days within 30 days of screening, had contralateral major amputation of the lower extremity, was unable to sustain offloading as defined by the protocol, allergy to glycerol or to the primary or secondary dressing materials used in the trial, was pregnant or nursing or a woman of child‐bearing potential who is unwilling to avoid pregnancy or use an appropriate form of birth control, or the index ulcer had been previously treated with cUC (BioTissue Holdings Inc., Miami, FL).

### 2.2 Treatment Procedures

Patients in both treatment groups received SOC throughout the duration of the study including debridement, wound cleansing using sterile saline, a non‐ionic cleanser or a hypochlorous solution, primary wound dressings including a non‐adherent, standard foam pad, with or without a hydrogel beneath the dressing, or an alginate dressing, a secondary retention bandage appropriate to the amount of wound exudate, and an off‐loading device appropriate to the location of the wound with a full‐length boot or total contact cast. Furthermore, all patients received osteomyelitis management, which consisted of thorough removal of infected and devitalised bone and soft tissue, biopsies of bone at baseline for histology and microbiologic testing, wound lavage, and a 6‐week course of systemic antibiotics based on culture and sensitivity results. Systemic antibiotics may have been given empirically at baseline, with adjustments made on the basis of culture and sensitivity results. If skin and soft tissue infection was present during the course of the study without evidence of osteomyelitis, a deep tissue biopsy was taken for histopathology, culture and sensitivity analysis was performed, and the antibiotic regimen adjusted (by starting a 2‐week course of antibiotics if none were being taken or antibiotic revision if still being taken for a previous infection). This SOC was adopted through the consensus of current clinical practice and review of the clinical literature for managing DFUs per guidelines developed by the Diabetes Panel of the American College of Foot and Ankle Surgeons, the Infectious Diseases Society of America for diagnosis and treatment of diabetic foot infections in 2012, and the Society for Vascular Surgery in collaboration with the American Podiatric Medical Association and the Society for Vascular Medicine in 2016 for managing DFUs presenting with clinical concerns of infection [4, 44, 45].

After all screening eligibility criteria were confirmed, the Initial Procedure Visit occurred on the same day or within 7‐days of screening. Wounds that were able to be closed by primary intention following debridement resulted in screen failure. Wounds that could not be closed by primary intention proceeded to randomisation using a computer‐based randomisation tool. For patients randomised to the treatment group, the test article, cUC, was secured with sutures, staples or both to cover the entire debrided wound surface at baseline.

**FIGURE 1:**
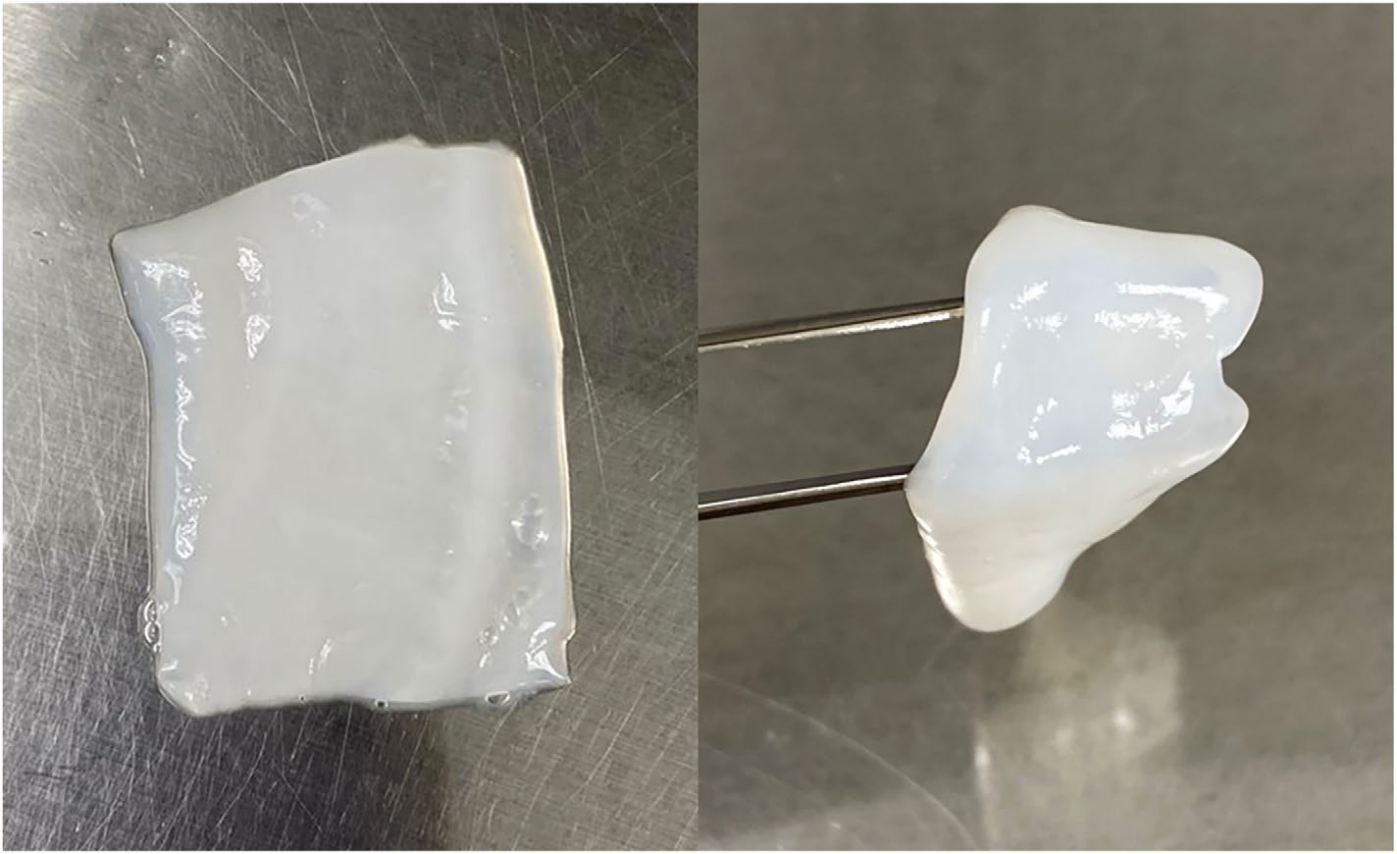
cUC allograft comprised of cryopreserved human amniotic membrane derived from the umbilical cord. It is available in multiple sizes and has a 2‐year shelf life stored under frozen conditions.

cUC (BioTissue Holdings Inc., Miami, FL) is derived from donated human placental tissue following healthy, live, caesarean section, full‐term births after determination of donor eligibility and placenta suitability. cUC is manufactured in compliance with both current good tissue practices (CGTP) and current good manufacturing practices (CGMP) utilising the proprietary CryoTek process, which devitalises all living cells but retains the natural structural and biological characteristics relevant to this tissue. cUC is manufactured in various sizes and is stored in a medium of lactated Ringer's/glycerol (1:1) under frozen conditions (Figure 1).

Patients in both treatment groups were evaluated weekly for safety and efficacy throughout the 16‐week treatment period. At each visit, the wound was debrided as necessary for patients in both groups, and all patients received dressing changes. For participants randomised to the treatment group, cUC could be re‐applied if healing was not evident or the product was dislodged, at no less than 4‐week intervals over the 16‐week treatment period, for a maximum of four total applications throughout the study. If skin and soft tissue infection (SSTI) was present, cUC application was withheld until clinical signs and symptoms were resolved. Furthermore, a deep tissue biopsy was obtained for histopathology, culture and sensitivity, and a 2‐week course of antibiotics was initiated if none were being taken (or antibiotics were revised if still being taken for a previous infection). For DFUs in the treatment arm that did show evidence of healing, additional applications of cUC were withheld on a week‐by‐week basis. If the wound did not reduce in area by ≥ 50% at 8 weeks versus the post‐debridement measurement at baseline, adjunctive therapies (i.e., silver‐containing products and collagen dressings) were permitted. Other treatments such as topical antibiotics, negative pressure wound therapy, hyperbaric oxygen, growth factors or cellular products and revascularisation procedures were restricted until Week 26.

### 2.3 Assessments and Endpoints

At each weekly visit, assessment of the foot and lower extremity, wound, concomitant medications, adverse events (AEs), and patient compliance regarding off‐loading was performed. Photographs of the wound were taken using the electronic wound imaging and measuring device (eKare inSight measuring device; eKare Inc., Fairfax, VA), which also provided automatic tracing of area (cm^2^), depth (cm) and volume (cm^3^) of the wound. Patients whose wounds closed at any time during the trial moved to a series of two consecutive confirmation of closure visits, 2 weeks apart, then continued with monthly observational visits until the end of the study. The determination of wound closure was made by the Investigator based on visual and tactile assessment of the wound. To reduce bias in the ascertainment of closure, the confirmation of wound healing was overseen by an independent blinded reviewer. Discordant opinions were adjudicated by a second independent blinded reviewer.

The intent‐to‐treat (ITT) population was the primary analysis population for clinical efficacy endpoints, which included all patients who received cUC or SOC after randomisation. The primary efficacy endpoint was time in days to complete wound healing within the first 26 weeks or 182 days, which was calculated as the number of days between the date of randomisation and the date of the initial visit where the wound was first observed to be closed. Complete wound healing was defined as complete epithelialisation, without drainage or the need for a dressing, confirmed at two consecutive visits, 2 weeks (no less than 14 days) apart. Secondary endpoints included the proportion of wounds that attained complete closure by Week 50 and the proportion of index ulcer complications by Week 50, including minor (portions of the foot) and major (ankle, above or below the knee) amputations. Additional outcomes included the proportion of wounds healed by Weeks 12 and 16 as well as wound healing outcomes by number of cUC applications.

### 2.4 Sample Size Calculations

The sample size for this trial was estimated using CreoStat HB Study Size software, v3.0.2. The sample size was estimated using a log rank, two‐sided, non‐parametric proportional hazards (Freedman) approach, with *α* set to 0.025, 1 − *β* set to 0.90, wound ‘survival’ in the control arm set to 60% (thus 40% healed), and wound ‘survival’ in the cUC arm set to 35% (thus 65% healed) based on the previously published open‐single arm study [43]. Assuming 1:1 randomisation and a 10% dropout, 94 patients per group were needed per group. As an added margin, 220 participants were enrolled.

### 2.5 Statistical Analysis

All analyses were performed for the ITT population, which included all enrolled patients who were randomised to treatment. All continuous data were expressed as mean ± standard deviation (range), and categorical variables were expressed as frequency and percentages. Pearson chi square was used to compare categorical data (ratios) between groups, and a two‐sample *T*‐test with Satterthwaite approximation was used to compare continuous data (means). The primary efficacy endpoint was summarised using the Kaplan–Meier method using the log‐rank test. The time to the closure of complete wound healing was considered censored at the corresponding time point for patients who discontinued from the treatment period prematurely, had missing data of wound closure over the confirmatory period, or whose index ulcers were not deemed to be completely closed at the end of the 26‐week treatment period. The median time to complete wound closure was presented along with confidence intervals (CIs), which were two‐sided with 95% coverage. Treatment groups were further stratified by baseline wound area (≤ 8 cm^2^ or > 8 cm^2^), and the proportion of healed wounds was compared using chi square tests. A Cox regression analysis (i.e., proportional hazards model) was also used to assess the effect of cUC treatment on time to complete wound healing compared to SOC in terms of hazard ratio (HR) and difference in the curve of the proportion of patients reaching complete wound healing over 26 weeks, adjusting for baseline ulcer depth, location, duration, recurrence, and presence of vascular disorders, and stratified by wound size (≤ 8 cm^2^ or > 8 cm^2^). Secondary analyses were reported as summary statistics and assessed using chi square test. Wound healing outcomes by number of cUC applications were also reported using a time‐to‐event analysis. A *p*‐value less than 0.05 was considered statistically significant.

## 3 Results

**FIGURE 2:**
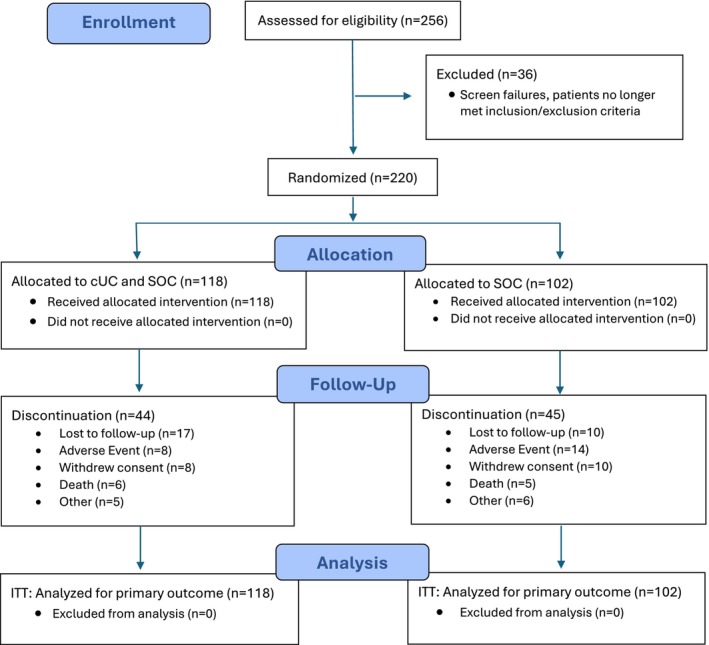
CONSORT flow diagram [46].

A total of 256 patients were screened during the 7‐day screening period, of which 36 patients were considered screen failures (Figure 2). Of the 220 eligible patients who were enrolled, 118 patients were randomised to the cUC group, and 102 patients were randomised to the SOC group. A total of 15 patients were lost to follow‐up or withdrew consent prior to Week 26 (7 SOC, 8 cUC), and 19 patients were lost to follow‐up or withdrew consent after Week 26 (13 SOC, 17 cUC). All 220 patients were included for the ITT analysis of both primary and secondary endpoints.

Patient characteristics and demographics were similar between groups, with no statistically significant differences observed between the cUC and SOC group (Table 1). Of the 220 patients in the ITT population, 92.7% (*n* = 204) had Type II diabetes, and the majority of patients (80.5%, *n* = 177) were male. The mean age was 57.1 ± 10.9 years (range: 31–84 years) at baseline, and 49.1% (*n* = 108) of patients were current or former smokers. The average body mass index (BMI) was 31.3 ± 6.7 kg/m^2^, with 53.2% (*n* = 117) of patients classified as obese. The vast majority of patients had neuropathy (*n* = 194, 88.2%), with 72.7% of patients (*n* = 160) reporting peripheral neuropathy and 15.5% of patients (*n* = 34) reporting diabetic neuropathy (Table 2). Furthermore, 87.3% of all patients (*n* = 192) reported a history of a vascular disorder, including hypertension (83.2%), peripheral vascular disease (29.5%) and peripheral arterial occlusive disease (9.1%). Renal and urinary disorders affected 42.7% of all patients (*n* = 94), including chronic kidney disease in 46 patients (20.9%). Lastly, cardiac disorders were reported in 72 patients (32.7%), with the most common being coronary artery disease (15.5%) and congestive heart failure (10%). Notably, 75 patients (34.1%) reported a prior toe amputation, and 53 patients (24.1%) reported a foot amputation (Table 2).

**TABLE 1:** Patient demographics of the ITT population.

	SOC (*n* = 102)	cUC (*n* = 118)	*p*
Age (years)	57.3 ± 11.6	56.9 ± 10.3	0.74
	58.0 (31, 84)	57.0 (32, 82)	
Gender			
Male	85 (83.3%)	92 (78.0%)	0.32
Female	17 (16.7%)	26 (22.0%)	0.32
Ethnicity			
Hispanic	38 (37.3%)	44 (37.3%)	0.99
Non‐Hispanic	64 (62.7%)	74 (62.7%)	0.99
Race			
White	79 (77.5%)	89 (75.4%)	0.72
Black or African American	15 (14.7%)	22 (18.6%)	0.44
Other	4 (3.9%)	3 (2.5%)	0.56
Asian	3 (2.9%)	3 (2.5%)	0.86
American Indian or Alaskan Native	1 (1.0%)	1 (0.8%)	0.92
Body mass index (kg/m^2^)	32.0 ± 6.6	30.6 ± 6.8	0.14
	31.9 (19.5, 54.0)	29.3 (17.9, 53.3)	
Smoker			
Current	16 (15.7%)	24 (20.3%)	0.37
Former	30 (29.4%)	38 (32.2%)	0.66
Never	56 (54.9%)	56 (47.5%)	0.27

*Note:* Continuous data presented as mean ± standard deviation; median (range) and categorical data presented as *n* (%) unless otherwise specified.

Abbreviations: cUC, cryopreserved umbilical cord; SOC, standard of care.

**TABLE 2:** Relevant medical history and prior procedures.

	SOC (*N* = 102)	cUC (*N* = 118)	Overall (*N* = 220)
Metabolism and nutrition disorders	102 (100)	118 (100)	220 (100)
Type 2 diabetes mellitus	97 (95.1)	107 (90.7)	204 (92.7)
Type 1 diabetes mellitus	4 (3.9)	9 (7.6)	13 (5.9)
Hyperlipidaemia	61 (59.8)	62 (52.5)	123 (55.9)
Dyslipidaemia	7 (6.9)	4 (3.4)	11 (5.0)
Nervous system disorders	92 (90.2)	108 (91.5)	200 (90.9)
Neuropathy peripheral	77 (75.5)	83 (70.3)	160 (72.7)
Diabetic neuropathy	15 (14.7)	19 (16.1)	34 (15.5)
Cerebrovascular accident	7 (6.9)	3 (2.5)	10 (4.5)
Vascular disorders	89 (87.3)	103 (87.3)	192 (87.3)
Hypertension	86 (84.3)	97 (82.2)	183 (83.2)
Peripheral vascular disease	25 (24.5)	40 (33.9)	65 (29.5)
Peripheral arterial occlusive disease	8 (7.8)	12 (10.2)	20 (9.1)
Deep vein thrombosis	6 (5.9)	3 (2.5)	9 (4.1)
Peripheral venous disease	3 (2.9)	2 (1.7)	5 (2.3)
Renal and urinary disorders	44 (43.1)	50 (42.4)	94 (42.7)
Chronic kidney disease	24 (23.5)	22 (18.6)	46 (20.9)
Acute kidney injury	7 (6.9)	8 (6.8)	15 (6.8)
End stage renal disease	5 (4.9)	10 (8.5)	15 (6.8)
Renal failure	3 (2.9)	2 (1.7)	5 (2.3)
Cardiac disorders	35 (34.3)	37 (31.4)	72 (32.7)
Coronary artery disease	14 (13.7)	20 (16.9)	34 (15.5)
Cardiac failure congestive	10 (9.8)	12 (10.2)	22 (10.0)
Atrial fibrillation	6 (5.9)	9 (7.6)	15 (6.8)
Myocardial infarction	7 (6.9)	3 (2.5)	10 (4.5)
Cardiac failure	4 (3.9)	4 (3.4)	8 (3.6)
Surgical and medical procedures	79 (77.5)	88 (74.6)	167 (75.9)
Toe amputation	32 (31.4)	43 (36.4)	75 (34.1)
Foot amputation	26 (25.5)	27 (22.9)	53 (24.1)
Leg amputation	0 (0)	2 (1.7)	2 (0.9)
Coronary artery bypass	8 (7.8)	8 (6.8)	16 (7.3)
Cholecystectomy	6 (5.9)	6 (5.1)	12 (5.5)
Coronary arterial stent insertion	3 (2.9)	5 (4.2)	8 (3.6)
Renal transplant	3 (2.9)	3 (2.5)	6 (2.7)

*Note:* Data presented in *n* (*n*/*N*, %) unless otherwise specified.

Baseline characteristics of the index ulcers were similar between groups, with no statistically significant differences observed between the cUC and SOC groups (Table 3). The mean baseline wound area for the cUC and SOC groups was 5.64 ± 5.5 cm^2^ and 5.30 ± 4.6 cm^2^, respectively. Percent wound granulation at baseline was 83.8% for the cUC group and 83.7% for the SOC group. The mean duration of the target ulcer as of the baseline visit was 233.1 ± 451.2 days and 185.5 ± 426.7 days for the cUC and SOC groups, respectively. Of the 220 ITT patients, 38 (17.3%) had recurrent index ulcers at baseline. The number of ulcers on the index foot for all patients in the ITT population was 1.3 on average (range: 1–4 ulcers), with 21.4% (*n* = 47) of patients having two or more ulcers. The majority of index ulcers in this study were located on the plantar surface of the foot (46.8%). While there was a larger proportion of plantar DFUs in the SOC group compared to the cUC group (52.9% vs. 41.5%), this difference was not statistically significant (*p* = 0.09).

**TABLE 3:** Baseline characteristics of index ulcers by treatment group.

	SOC (*N* = 102)	cUC (*N* = 118)	*p*
Ulcer area (cm^2^)	5.30 ± 4.6	5.64 ± 5.5	0.61
	3.50 (0.3, 17.0)	3.75 (0.1, 29.9)	
Ulcer depth (cm)	0.82 ± 0.69	0.86 ± 0.69	0.65
	0.60 (0.0, 3.7)	0.70 (0.0, 2.6)	
Ulcer volume (cm^3^)	3.24 ± 4.2	3.73 ± 5.7	0.47
	1.20 (0.0, 16.0)	1.20 (0.0, 34.4)	
Wound granulation (%)	83.7 ± 21.8	83.8 ± 19.4	0.99
	93.0 (0, 100)	92.0 (13, 100)	
Ulcer duration (days)	185.5 ± 426.7	233.1 ± 451.2	0.42
	75.5 (3, 3905)	98.5 (11, 4019)	
Ulcer location			
Heel	5 (4.9%)	12 (10.2%)	0.14
Lateral surface	18 (17.6%)	19 (16.1%)	0.76
Medial surface	9 (8.8%)	12 (10.2%)	0.73
Plantar surface	54 (52.9%)	49 (41.5%)	0.09
Toes (interdigital)	16 (15.7%)	21 (17.8%)	0.68
Other	0 (0.0%)	5 (4.2%)	0.036
Number of ulcers on index foot	1.3 ± 0.6	1.3 ± 0.5	0.79
	1.0 (1, 3)	1.0 (1, 4)	
Recurrent ulcer	20 (19.6%)	18 (15.3%)	0.39

Abbreviations: cUC, cryopreserved umbilical cord; SOC, standard of care.

Following initial debridement at baseline, three DFUs in the cUC group and 15 DFUs in the SOC group had the wound partially closed with sutures affecting wound size (*p* = 0.001). By the primary end point (26 weeks), 11/15 (73.3%) of these DFUs in the SOC group healed, and two‐third (66.6%) of DFUs in the cUC group healed. Median time to wound closure was 54 days (range: 20–135).

A total of 139 patients achieved complete healing in the ITT population within 26 weeks (78 patients or 66.1% in the cUC group and 61 patients or 59.8% in the SOC group; *p* = 0.40). The median time to heal in days for the cUC and SOC groups was 106 days (95% CI: 84–119 days) and 104 days (95% CI: 84–121 days), respectively (*p* = 0.99). Cox proportional hazards regression analysis showed that cUC treatment increased the probability of complete wound closure by 18% compared with SOC (HR = 1.18, 95% CI: 0.83–1.68, *p* = 0.35). When assessing wounds ≤ 8 cm^2^, 73.6% (*n* = 64) of patients in the cUC group and 71.1% (*n* = 54) of patients in the SOC group demonstrated complete wound closure by 26 weeks (*p* = 0.72). For those wounds > 8 cm^2^, the cUC group demonstrated a higher rate of wound closure than the SOC group (45.2% vs. 26.9%, respectively) at 26 weeks, although this difference was not statistically significant (*p* = 0.16).

At 12 weeks, complete wound closure was attained in 46 (39.0%) patients in the cUC group compared to 37 (36.3%) patients in the SOC group (*p* = 0.68). The rate of complete wound healing further increased at 16 weeks to 49.2% (*n* = 58) and 47.1% (*n* = 48) in the cUC and SOC groups, respectively (*p* = 0.76). By the end of the study, or 50 weeks, 77.1% (*n* = 91) of patients treated with cUC achieved complete healing compared to 71.6% (*n* = 73) of patients treated with SOC (*p* = 0.29). There were no significant differences in healing rates between the two treatment arms at any timepoint.

**FIGURE 3:**
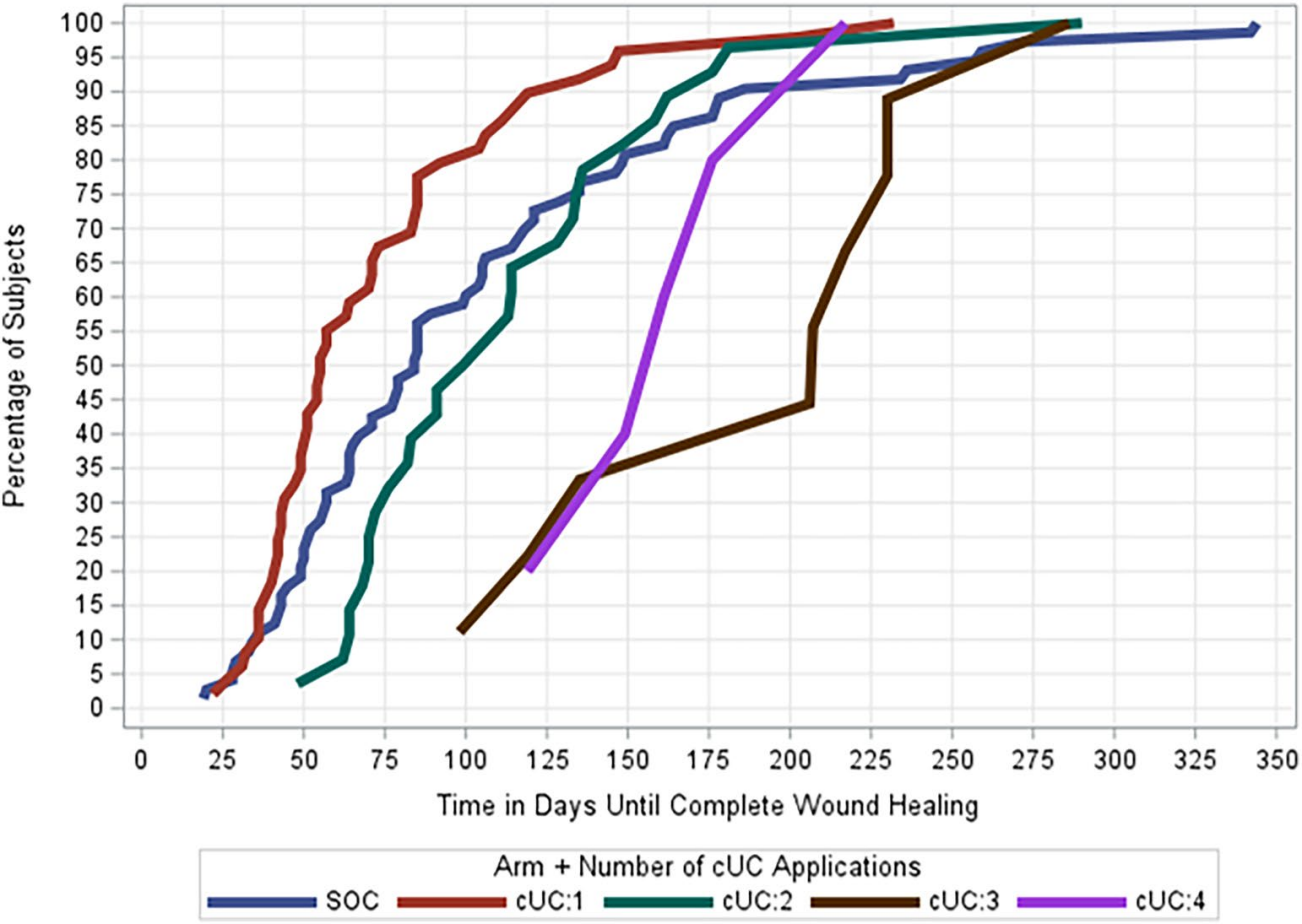
Time to complete wound closure in the ITT population among DFUs that healed by number of cUC treatment applications.

**FIGURE 4:**
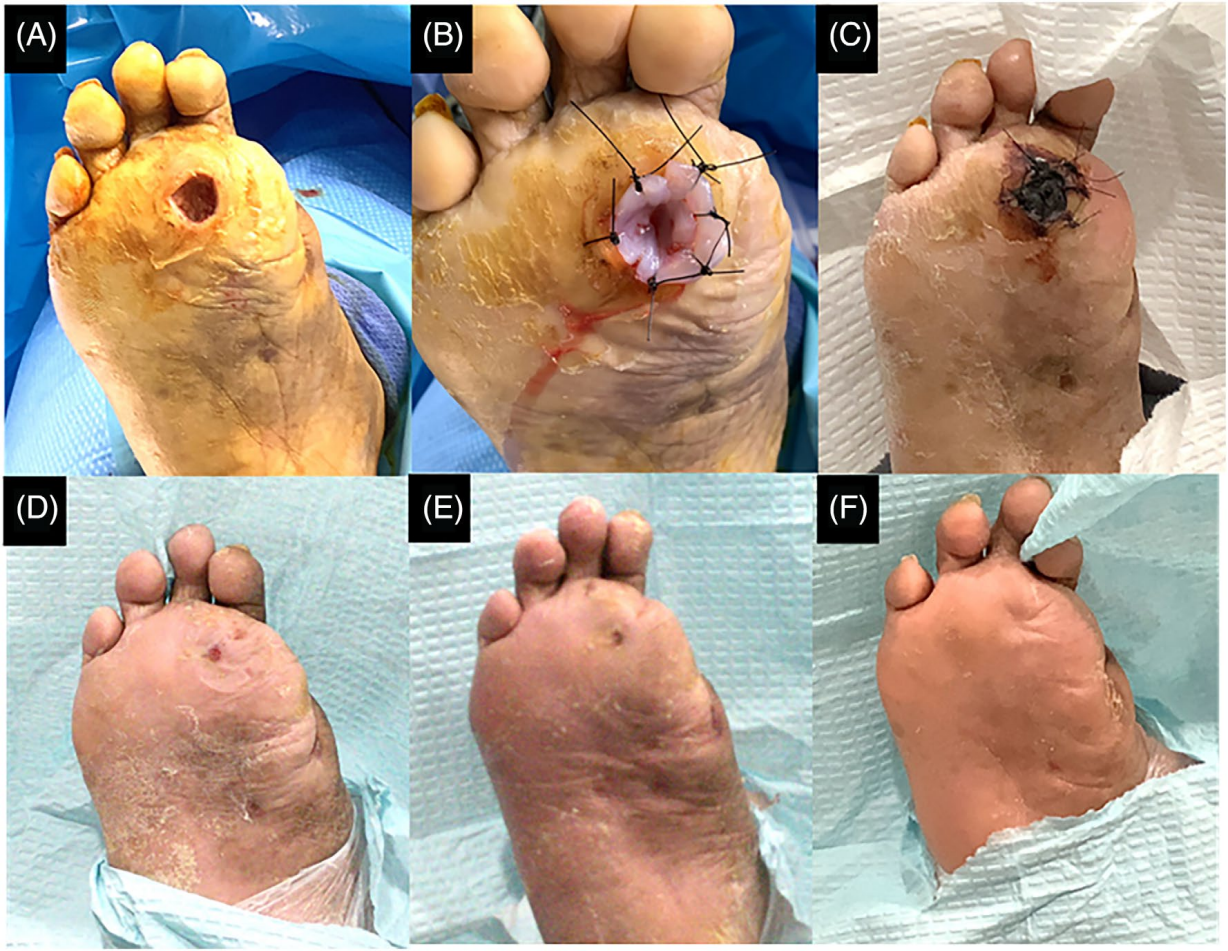
Representative case example of successful wound closure following single application of cryopreserved umbilical cord. A 65‐year‐old Hispanic male (BMI: 36.12 kg/m^2^) with peripheral vascular disease, bilateral neuropathy and prior amputation of the hallux presented with a DFU on the plantar surface of the foot, which measured 1.9 cm^2^ post‐debridement (A). Following debridement, a single application of cryopreserved umbilical cord was fixated to the wound bed with sutures (B). Patient returned at Day‐7 with a ‘black cap’ over the index ulcer (C), which was still evident at Day‐49. At Day‐55, the ‘black cap’ had fallen off and the wound measured 0.2 cm^2^ (D). At the following visit or Day‐63, the DFU exhibited complete wound closure (E), which remained healed without recurrence by the end of study visit or Day‐343 (F).

Patients in the treatment group received an average of 1.86 ± 1.0 (range: 1–4) cUC applications throughout the 16‐week treatment period. Specifically, 56 (25.5%) patients received a single cUC application, 36 (16.4%) patients received two applications, 12 (5.5%) patients received three applications and 14 (6.4%) patients received four total applications. Time to achieve complete wound healing by number of treatment applications is presented in Figure 3. An average of 1.67 ± 0.87 applications was required to achieve wound closure in the cUC group. A representative case example is shown in Figure 4.

There were 33 patients who had an index ulcer complication while enrolled in the study (14 patients, 11.9% in the cUC group vs. 19 patients, 18.6% in the SOC group; *p* = 0.16). This included 22 patients (9 patients or 7.6% in the cUC group and 13 patients or 12.7% in the SOC group, *p* = 0.21) who had a major or minor amputation of the index limb while enrolled in the study. The time to major/minor amputation ranged from 13 to 304 days in the SOC group and 10 to 310 days in the cUC group (*p* = 0.18). Of these patients, 11 had a major amputation (six patients, 5.1% in the cUC group and five patients, 4.9% in the SOC group; *p* = 0.95). Overall safety was comparable between groups, with 89.8% of subjects in the cUC group and 87.3% of subjects in the SOC group experiencing an AE throughout the 50‐week study period. There were no significant differences in AEs between the two treatment arms. Two patients (1.7%) experienced a treatment emergent AE that was considered related to the cUC, which included stitch abscess (*n* = 1) and muscle spasms (*n* = 1).

## 4 Discussion

This prospective, multi‐centre, RCT of 220 patients demonstrates that adjunctive use of cUC with SOC is safe and helps achieve a high healing rate (66.1% in 26 weeks) comparable to SOC alone (59.8% in 26 weeks) for late stage, complex, non‐healing DFUs with medically controlled osteomyelitis. Historically, RCTs investigating the efficacy of human cellular and tissue‐based products for DFUs have been limited to more superficial wounds without exposure of bone, tendon, muscle and/or joint capsule [47, 48, 49, 50, 51, 52, 53]. Furthermore, nearly all RCTs in wound care exclude patients with significant comorbidities to evaluate the efficacy of the study product, resulting in the ineligibility of more than half the wound care patient population [54]. For instance, many RCTs assessing wound coverings for DFUs have historically excluded patients with impaired kidney function (e.g., renal disease) [47, 48, 51, 55, 56, 57, 58, 59], hepatic function (e.g., liver failure) [47, 58, 60], or impaired vasculature (e.g., peripheral vascular disease, arterial insufficiency) [47, 48, 55, 57, 58] as well as those DFUs with exposure of either bone, muscle, tendon and/or joint capsule [47, 48, 51, 55, 56, 59, 61, 62, 63, 64]. The present study, however, included more severe, complex DFUs as evidenced by ‘depth’ showing exposed bone, tendon, muscle and/or joint capsule and a number of co‐existing health conditions including peripheral or diabetic neuropathy (88%), vascular disorders (87%), renal and urinary disorders (43%) and cardiac disorders (33%). Furthermore, 34% of patients had a prior toe amputation, and 24% of patients had a prior foot amputation. Collectively, the present study assessed the utility of cUC in a highly morbid population that has been largely excluded in prior RCTs and yet is highly prevalent within the United States [65].

Despite the severity of these wounds and the high morbidity of this patient population, 66.1% and 59.8% of DFUs in the present study attained confirmed wound closure by 26 weeks in the cUC and SOC groups, respectively. While the rate of complete wound closure in the cUC group was not significantly higher than that of the SOC group, the healing rates in the cUC group are comparable to previous studies assessing cUC for complex wounds including a single‐arm, prospective trial (16/32 or 50% closure by Week‐16) [43] as well as real‐world, retrospective data (26/33 or 79% in 16 ± 9.3 weeks [range: 4–44]) [41]. In the present study, 39% of complex DFUs in the cUC group healed by 12 weeks, and 49% healed within 16 weeks, which is comparable with the healing rates observed in the two aforementioned studies. These healing rates are also similar to other RCTs that assessed the use of wound coverings for both superficial and complex DFUs (Wagner 1 and 2 DFUs) at 12 weeks (33%–46%) [49, 56, 57, 63, 64, 66, 67] and 16 weeks (25%–52%) [57, 60, 63, 66, 68, 69]. Notably, 83.9% and 69.4% of wounds treated with one or two cUC applications healed by Week 26, respectively. In total, an average of 1.67 ± 0.87 applications were required to achieve complete wound closure by the end of the study in the cUC group. The low number of cUC applications is consistent with previous studies assessing cUC for complex DFUs, which reported a mean of 1.5 ± 0.8 applications [43] and 1.2 ± 0.4 cUC applications [41] to attain wound closure. This number is far lower than that reported in many other studies, which allowed weekly graft application of advanced tissue products throughout the treatment period. In fact, many RCTs have reported the use of six graft applications or more on average, with some studies reporting up to nine applications throughout a 12–16‐week treatment period [47, 50, 53, 56, 58, 63, 68, 70]. Thus, the relatively low number of cUC applications with SOC may result in lower treatment costs compared to other advanced skin substitutes to attain similar wound closure rates.

The healing rate observed in the SOC group is relatively higher than in other reported studies. In a RCT that assessed the use of recombinant human epidermal growth factor versus SOC in a similar patient population with osteomyelitis, 52.1% of DFUs in the SOC group healed at 52‐weeks [71]. In our study, 71.6% of DFUs in the SOC group healed by 50 weeks. We suspect that such a discrepancy might be attributed to the 6‐week course of systemic broad‐spectrum antibiotics in our study as opposed to being not required for all patients to clear infections before initiating treatment in that study [71]. Additionally, our study undertook rigorous surgical measures in managing osteomyelitis at baseline, which consisted of debridement to remove infected and devitalised bone and soft tissue (including bone resection), wound lavage, and biopsies of bone at baseline for histology and microbiologic and drug sensitivity testing. The healing rate of the SOC at 12 weeks was 36.3% in our study, which was slightly higher than that of a number of RCTs, which studied less severe wounds (without exposure of bone, tendon or joint capsule) [53, 55, 66, 67, 72]. In one RCT [57], 38% and 49% of patients treated with SOC healed at 16 and 24 weeks compared to 47% and 60% in the present study, respectively. We believe that another important factor that may explain the aforementioned discrepancy in the wound healing rate with SOC is the significantly higher proportion of DFUs in the SOC group that had the wound partially closed with sutures at baseline after randomisation in our study. By excluding this subset of patients in the SOC group, the overall healing rate for SOC would have been lower, although not statistically significant due to the sample size. Nonetheless, the study results are impactful as they demonstrate that consistent, high‐quality SOC alone can lead to favourable outcomes in complex DFUs, including those with controlled osteomyelitis.

The use of cUC in conjunction with SOC was safe as evidenced by comparable AE rates between the two treatment groups. Furthermore, index ulcer complications, including major and minor amputations, were lower in the cUC group at 50 weeks although not statistically significant. By the end of the study (50 weeks), 7.6% of patients in the cUC group had a major or minor amputation compared to 12.7% in the SOC group. While this outcome measure wasn't sufficiently powered to detect a statistically significant difference, these amputation rates are lower than previous studies that utilised SOC (19%–25%) [71, 73, 74], highlighting the usefulness of adopting rigorous measures in managing infection throughout the study period as described in the present study. Collectively, the results of the present study are significant given the unmet clinical need for a treatment for complex DFUs with exposed underlying tendon, muscle, ligament and bone, which is associated with a high risk of complications, including amputation [12, 16, 17, 18, 19, 20].

There are some limitations in the present study. Due to the nature of the product and the treatment modality employed, neither the investigators nor patients could be blinded. Nevertheless, the risk of bias is largely overcome by the objective nature of the endpoint measurement, the use of multiple investigative sites, and the additional use of blinded, third‐party reviewers. Off‐loading compliance, which has been shown to be significantly associated with improved wound healing outcomes [75], was not assessed through diaries but rather through the observation of the off‐loading device at each visit. Additionally, re‐application of cUC, as well as debridement was left to the discretion of the investigator at each visit, which could have resulted in treatment variances among sites. Lastly and more importantly, significantly more DFUs in the SOC group received partial wound closure with sutures at baseline compared to the cUC group, which may explain the high healing rate in the SOC group. Nevertheless, there are notable strengths with this study. This study had a long‐term follow‐up period out to 50 weeks, had a patient population that is representative of what is seen in a real‐world clinical setting, and included all patients for analysis, giving this study high external validity. While cUC was not superior to SOC at any assessed time point, this RCT demonstrates that adjunctive cUC is safe and results in high rates of complete wound closure at 50 weeks.

## Conflicts of Interest

The authors declare no conflicts of interest.

## Data Availability

The data that support the findings of this study are available on request from the corresponding author. The data are not publicly available due to privacy or ethical restrictions.
